# Increased Circulating Th1 and Tfh1 Cell Numbers Are Associated with Disease Activity in Glucocorticoid-Treated Patients with IgG4-Related Disease

**DOI:** 10.1155/2020/3757015

**Published:** 2020-11-27

**Authors:** Changsheng Xia, Caoyi Liu, Yanying Liu, Yan Long, Lijuan Xu, Chen Liu

**Affiliations:** ^1^Department of Clinical Laboratory, Peking University People's Hospital, Beijing, China; ^2^Institute of Blood Transfusion, Chinese Academy of Medical Sciences, Chengdu, China; ^3^Department of Rheumatology and Immunology, Peking University People's Hospital, Beijing, China; ^4^Department of Immunology, School of Basic Medicine Sciences, Peking University Health Science Center, Beijing, China

## Abstract

**Background:**

This study is aimed at exploring the changes and significance of circulating Th and Tfh cell subsets in glucocorticoid-treated IgG4-RD patients.

**Methods:**

39 glucocorticoid-treated IgG4-RD patients and 22 healthy controls (HC) were enrolled. Peripheral blood mononuclear cells were separated, and circulating Th and Tfh cell subsets were examined by flow cytometry according to the surface and intranuclear markers. Disease activity was accessed by the IgG4-RD responder index (RI) score. Correlation analyses were conducted between Th/Tfh subset numbers and clinical indicators. The receiver operating characteristic (ROC) curve was used to evaluate the efficacy of Th and Tfh subsets to distinguish active IgG4-RD patients from remission IgG4-RD patients.

**Results:**

Circulating Th1, Th17, Tfh1, and Tfh17 cells were significantly increased in active IgG4-RD patients compared with HC. Th1 and Tfh1 numbers were positively correlated with serum IgG4 levels in patients with IgG4-RD. Meanwhile, the absolute numbers of circulating Th1 and Tfh1 cells were positively correlated with IgG4-RD RI scores. The areas under the curve (AUC) were 0.8276 for Th1 and 0.7310 for Tfh1, 0.5862 for Tfh2, and 0.6810 for Tfh17.

**Conclusion:**

Increased circulating Th1 and Tfh1 subsets are related to elevated serum IgG4 levels in active IgG4-RD patients during glucocorticoid treatment, which may play an important role in the course of IgG4-RD disease, and could be potential biomarkers for monitoring disease activity of IgG4-RD.

## 1. Introduction

IgG4-related disease (IgG4-RD) is a chronic immune-mediated fibroinflammatory disorder characterized by tumefactive lesions, a dense lymphoplasmacytic infiltration rich in IgG4-positive plasma cells, storiform fibrosis, and frequently elevated serum IgG4 concentrations [1]. The majority of infiltrating cells are small lymphocytes that are composed predominantly of T cells distributed diffusely throughout the lesion and intermingled with plasma cells [2]. Infiltrating T cells in the lesion are primarily activated CD4^+^ T cells, which play an important role in the course of IgG4-RD [3].

Previous studies have reported that CD4^+^ cytotoxic T lymphocytes (CTLs) and follicular helper T (Tfh) cells that infiltrated tissue lesions were the main CD4^+^ T cells at disease sites in IgG4-RD [4–8]. Circulating CD4^+^ CTLs and Tfh cells were found to be expanded in patients with IgG4-RD [5, 6, 9]. Circulating CD4 T helper (Th) cells can be divided into Th1 cells (CXCR3^+^CCR6^−^), Th2 plus naïve CD4^+^ cells (CXCR3^−^CCR6^−^, CXCR3*^−^*CCR6^−^CCR4^+^ cells represent Th2, and CXCR3^−^CCR6^−^CCR4^−^ cells belong to naïve CD4), and Th17 cells (CXCR3^−^CCR6^+^) [10, 11]. CD4^+^ CTLs, which express the transcription factors of ThPOK, Runx3, and T-bet, are mainly differentiated from Th1 cells [12, 13]. Accordingly, the expansion of circulating CD4^+^ CTLs may lead to elevated Th1 cells in the peripheral blood of IgG4-RD patients. Tfh cells represent a distinct CD4^+^ T cell subset providing key signals to B cells for their differentiation into plasma cells which secrete high-affinity antibodies in the germinal center [14, 15]. Peripheral blood CD4^+^CXCR5^+^ T cells are considered to be a circulating pool of memory Tfh cells, which can be divided into Tfh1 (CXCR3^+^CCR6^−^), Tfh2 (CXCR3^−^CCR6^−^), and Tfh17 (CXCR3^−^CCR6^+^) cells, and they have different capabilities to help the differentiation of B cell subsets [16, 17]. Some studies have investigated the role of circulating CD4^+^ T cell subsets in IgG4-RD. However, most previous studies have focused on untreated IgG4-RD patients. Few studies have explored the relationship between circulating CD4^+^ T cell subsets and disease activity in treated IgG4-RD patients.

In this study, we aim to determine the relationship between circulating Th and Tfh cell subsets and serum IgG4 levels as well as disease activity in glucocorticoid-treated IgG4-RD patients. We intend to clarify the changes and clinical significance of circulating Th and Tfh cell subsets in treated IgG4-RD patients.

## 2. Results

### 2.1. Clinical Characteristics of the Patients with IgG4-RD

We involved 39 patients with IgG4-RD including the diagnosis of definite (*n* = 25), possible (*n* = 12), and probable (*n* = 2) IgG4-RD diagnosis results, and the clinical and demographic characteristics of the patients are described in Supplementary Table 1. Of those subjects, 38 (97.4%) were presented with ≥2 organs involved. Frequent sites of initial organ involvement included the submandibular glands (13 cases, 33.3%), lacrimal glands (7 cases, 17.9%), parotid gland (1 case, 2.6%), pancreas (10 cases, 25.6%), bile ducts (1 case, 2.6%), lymph nodes (2 cases, 5.1%), retroperitoneum (3 cases, 7.7%), sinus (1 case, 2.6%), and mesentery (1 case, 2.6%). The median RI score of these IgG4-RD patients was 5. These patients were divided into active (*n* = 29) and remission (*n* = 10) IgG4-RD patients according to RI scores.

We measured and compared levels of IgG4, IgG, IgE, C3, C4, and CRP in the serum of active IgG4-RD patients, remission IgG4-RD patients, and HC. As shown in Supplementary Figure 1, the concentrations of serum IgG4 were significantly higher in active IgG4-RD patients than in HC and remission patients (3.01 g/L versus 0.46 g/L and 0.85 g/L; *P* < 0.0001 and *P* = 0.0153, respectively). Similarly, IgE was significantly elevated in active IgG4-RD patients compared with HC and remission patients (103.40 IU/mL versus 24.94 IU/mL and 38.13 IU/mL; *P* = 0.0001 and *P* = 0.0483, respectively). In contrast, serum C4 levels were significantly decreased in patients with active IgG4-RD compared with HC (0.209 g/L versus 0.268 g/L, *P* = 0.0032). We also analyzed levels of lymphocytes and CD4^+^ T cell percentages and found elevated lymphocyte concentrations and CD4^+^ T cell percentages in active IgG4-RD patients compared to HC or remission patients (not shown).

### 2.2. Increased Th1 and Th17 Cells in Patients with Active IgG4-RD

**Figure 1:**
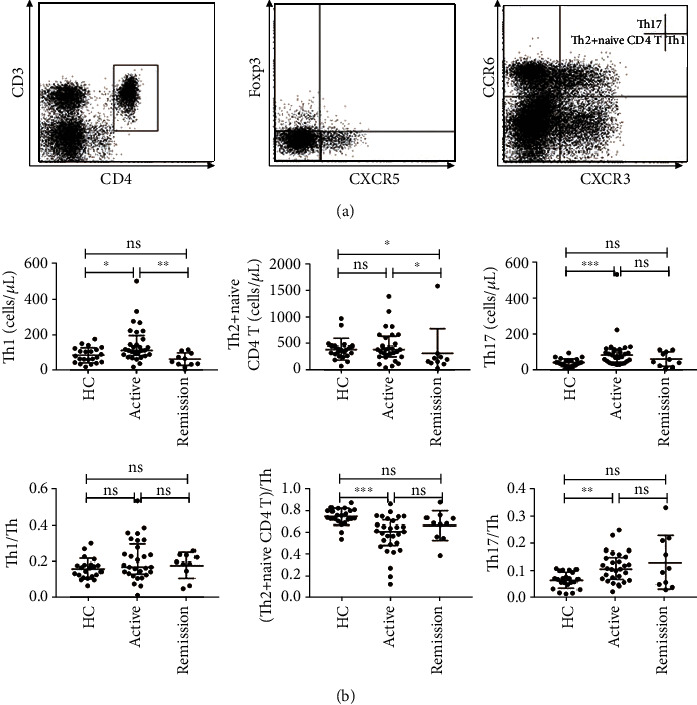
Analyses of Th cell subsets in active IgG4-RD patients, remission IgG4-RD patients, and healthy controls. Th and Tfh cells in PBMCs were analyzed by FCM. Circulating Th cells were defined as CD3^+^CD4^+^CXCR5^−^Foxp3^−^ cells. Th1, Th2 plus naïve CD4, and Th17 cell subsets were defined as CXCR3^+^CCR6^−^ cells, CXCR3^−^CCR6^−^ cells, and CXCR3^−^CCR6^+^ cells within Th cells. Circulating Tfh cells were defined as CD3^+^CD4^+^CXCR5^+^Foxp3^−^ cells. Tfh1, Tfh2, and Tfh17 cell subsets were defined as CXCR3^+^CCR6^−^ cells, CXCR3^−^CCR6^−^ cells, and CXCR3^−^CCR6^+^ cells among Tfh cells. (a) Representative dot plots of flow cytometry analysis and Th1, Th2 plus naïve CD4, and Th17 subsets were shown. (b) The absolute numbers (per *μ*L) and frequencies of circulating Th cell subsets in active IgG4-RD patients (*n* = 29), remission IgG4-RD patients (*n* = 10), and HC (*n* = 22). The error bars represented the median and interquartile range. The Mann–Whitney test was used to compare the subset levels. ns, *P* ≥ 0.05 (not significant); ^∗^*P* < 0.05; ^∗∗^*P* < 0.01; ^∗∗∗^*P* < 0.001.

We next measured Th cell levels in IgG4-RD patients and HC according to surface markers. Circulating Th cells were defined as CD3^+^CD4^+^CXCR5^−^Foxp3^−^ cells because we want to exclude the influence of Foxp3^+^ regulatory T cells. Th1, Th2 plus naïve CD4, and Th17 cell subsets were defined as CXCR3^+^CCR6^−^ cells, CXCR3^−^CCR6^−^ cells, and CXCR3^−^CCR6^+^ cells within the CD3^+^CD4^+^CXCR5^−^Foxp3^−^ Th cells, respectively. As shown in Figure 1, the absolute number (per *μ*L) of circulating Th1 cells was significantly increased in active IgG4-RD patients compared with HC and remission patients (111.9 cells/*μ*L versus 76.4 cells/*μ*L and 57.4 cells/*μ*L; *P* = 0.0376 and *P* = 0.0047, respectively). Moreover, the absolute numbers (per *μ*L) and frequencies of circulating Th17 cells were significantly higher in patients with active IgG4-RD than in HC (76.4 cells/*μ*L versus 30.6 cells/*μ*L, 0.107 versus 0.070; *P* = 0.0007 and *P* = 0.0067, respectively). Interestingly, the absolute numbers (per *μ*L) of circulating Th2 plus naïve CD4 cells were significantly lower in remission IgG4-RD patients than in active patients and HC (197.9 cells/*μ*L versus 400.9 cells/*μ*L and 374.6 cells/*μ*L; *P* = 0.0264 and *P* = 0.0474, respectively). The frequencies of circulating Th2 plus naïve CD4 cells were significantly lower in active IgG4-RD than in HC (0.611 versus 0.745; *P* = 0.0007).

### 2.3. Tfh1 and Tfh17 Cells in Patients with Active IgG4-RD Were Increased

**Figure 2:**
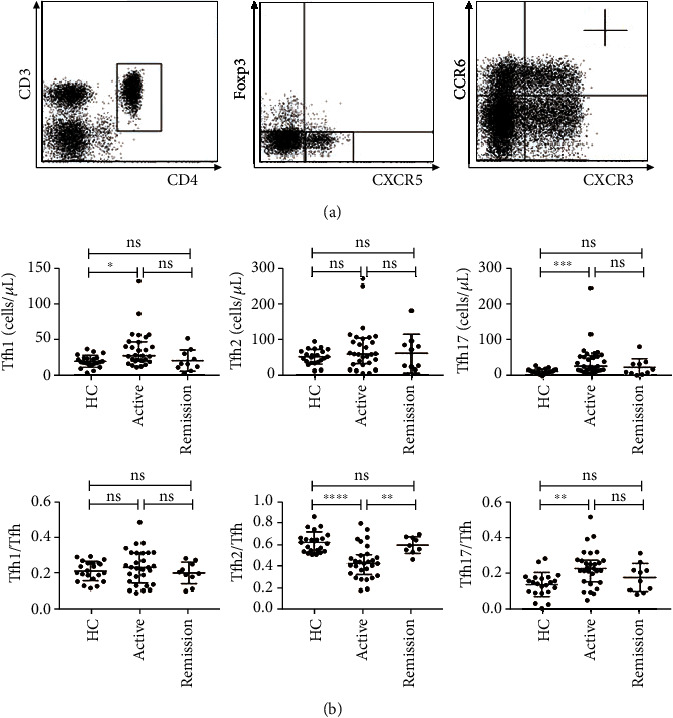
Tfh cell subset analysis in active IgG4-RD patients, remission IgG4-RD patients, and healthy controls. (a) Representative dot plots for FCM analysis. Tfh1, Tfh2, and Tfh17 were defined as CD4^+^CXCR5^+^Foxp3^−^CXCR3^+^CCR6^−^ cells, CD4^+^CXCR5^+^Foxp3^−^CXCR3^−^ CCR6^−^ cells, and CD4^+^CXCR5^+^Foxp3^−^CXCR3^−^CCR6^+^ cells, respectively. (b) The absolute numbers (per *μ*L) and frequencies of circulating Tfh subsets in active IgG4-RD patients (*n* = 29), remission IgG4-RD patients (*n* = 10), and HC (*n* = 22). The error bars represented the median and interquartile range. The Mann–Whitney test was used to compare the subset levels. ns, *P* ≥ 0.05 (not significant); ^∗^*P* < 0.05; ^∗∗^*P* < 0.01; ^∗∗∗^*P* < 0.001; ^∗∗∗∗^*P* < 0.0001.

We next analyzed circulating Tfh cell changes in these IgG4-RD patients. Circulating Tfh cells were defined as CD3^+^CD4^+^CXCR5^+^Foxp3^−^ cells. Foxp3^+^ cells were also excluded because we want to exclude the influence of follicular regulatory T cells. Tfh1, Tfh2, and Tfh17 cell subsets were defined as CXCR3^+^CCR6^−^ cells, CXCR3^−^CCR6^−^ cells, and CXCR3^−^CCR6^+^ cells within CD3^+^CD4^+^CXCR5^+^Foxp3^−^ Tfh cells, respectively. As shown in Figure 2, the absolute number (per *μ*L) of circulating Tfh1 cells was significantly increased in active IgG4-RD patients compared with HC (26.2 cells/*μ*L versus 17.4 cells/*μ*L; *P* = 0.0172). Moreover, the absolute numbers (per *μ*L) and frequencies of circulating Tfh17 cells were also significantly increased in patients with active IgG4-RD compared with HC (25.8 cells/*μ*L versus 10.4 cells/*μ*L, 0.226 versus 0.145; *P* = 0.0009 and *P* = 0.0027, respectively). Conversely, the frequencies of circulating Tfh2 cells were significantly decreased in active IgG4-RD patients compared with HC and remission patients (0.424 versus 0.612 and 0.572; *P* < 0.0001 and *P* = 0.0091, respectively).

### 2.4. Circulating Th1 Cells Are Positively Correlated with IgG4 Levels and RI Scores in IgG4-RD Patients

**Figure 3:**
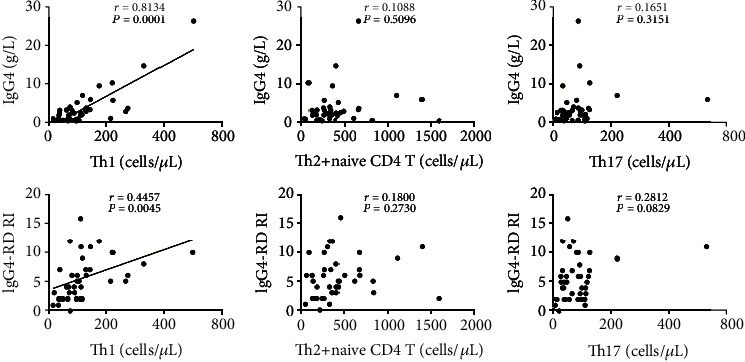
Correlation analysis of circulating Th cell subsets with serum IgG4 and IgG4-RD RI in IgG4-RD patients. Serum IgG4 concentrations of all IgG4-RD patients (*n* = 39) were measured. Correlation analyses were conducted between absolute numbers (per *μ*L) of Th subsets and serum IgG4 (up) or IgG4-RD RI scores (down) in IgG4-RD patients. Each plot represented the data of a patient. The Spearman test's *r* and *P* values for each parameter were listed. The *r* values were Spearman's correlation coefficients. For *P* values less than 0.05, data were presented as scatter plots with a linear fit.

**Figure 4:**
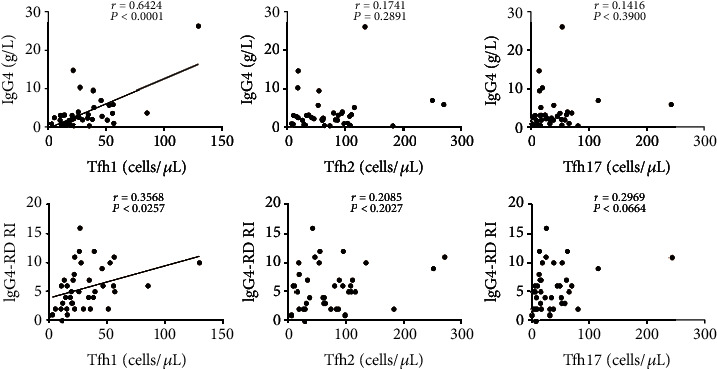
The correlations of circulating Tfh cell subsets with serum IgG4 and IgG4-RD RI in IgG4-RD patients. Correlation analyses were conducted between absolute numbers (per *μ*L) of Tfh subsets and serum IgG4 levels (up) or IgG4-RD RI scores (down) in IgG4-RD patients (*n* = 39). Each plot represented the data of one patient. The Spearman test's correlation coefficient *r* and *P* values were listed, and the *P* values less than 0.05 were linearly regressed to show relevant trends.

To determine the associations of Th cell subsets with clinical indicators, the correlations of Th cell subsets with serum IgG4 and RI scores in IgG4-RD patients were conducted. As shown in Figure 3, the absolute number (per *μ*L) of Th1 cells was found to be positively correlated with serum IgG4 levels and IgG4-RD RI scores (*r* = 0.8134 and 0.4457; *P* < 0.0001 and *P* = 0.0045, respectively). Similarly, the correlations of Tfh cell subsets with serum IgG4 and RI scores in IgG4-RD patients were also analyzed, respectively (Figure 4). Interestingly, we found that the number of Tfh1 cells was also positively correlated with serum IgG4 levels and IgG4-RD RI scores (*r* = 0.6424 and 0.3568; *P* < 0.0001 and *P* = 0.0257, respectively).

### 2.5. Th1 and Tfh1 Cell Numbers Can Be Used as Potential Biomarkers for IgG4-RD Disease Activity Monitoring

**Figure 5:**
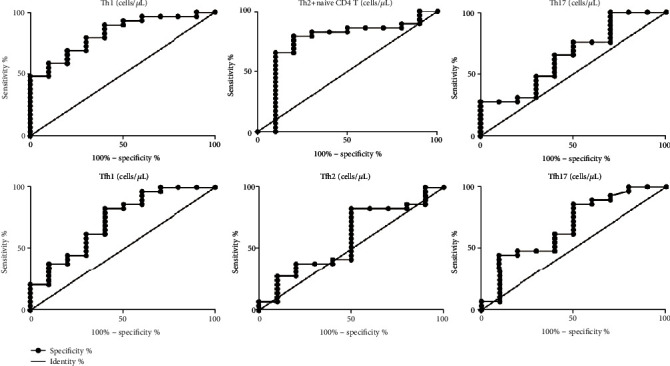
ROC analysis of the values of Tfh and Th subsets in the diagnosis of active IgG4-RD. Th (up) and Tfh (down) subset levels in 39 cases of IgG4-RD patients were analyzed, including 29 active and 10 remission IgG4-RD patients. The receiver operating characteristic (ROC) curves were performed to evaluate the efficacy of Th or Tfh subsets to distinguish active IgG4-RD from remission IgG4-RD patients. The areas under the curve (AUC) were 0.8276 for Th1 cells, 0.7586 for Th2 plus naïve CD4 cells, and 0.6517 for Th17 cells, and AUC were 0.7310 for Tfh1 cells, 0.5862 for Tfh2 cells, and 0.6810 for Tfh17 cells.

Since Th1 and Tfh1 subset numbers are significantly increased in active IgG4-RD patients and have significant positive correlations with disease activity, we further explored whether they could be used as potential markers for IgG4-RD disease activity monitoring. As shown in Figure 5, we used Tfh1 and Th1 cell numbers to distinguish active IgG4-RD from remission IgG4-RD and generated ROC curves. The areas under the curve (AUC) were 0.8276 for Th1 cells and 0.7310 for Tfh1 cells. We also analyzed the efficacy of using other cell subsets for diagnosis. The areas under the curve (AUC) were 0.7586 for Th2 plus naïve CD4 cells and 0.6517 for Th17 cells, and AUC were 0.5862 for Tfh2 cells and 0.6810 for Tfh17 cells (Figure 5).

## 3. Discussion

In this research, we studied the changes and significance of circulating Th and Tfh cell subsets in glucocorticoid-treated IgG4-RD patients. We found that circulating Th1 and Tfh1 subsets were increased and were related to serum IgG4 levels and RI scores in active IgG4-RD patients, which may play an important role in IgG4-RD, and could be potential biomarkers for monitoring disease activity of IgG4-RD during treatment.

IgG4-RD is often featured by elevated levels of serum IgG4 [18, 19]. Serum IgE concentrations are also increased in some patients [20]. In the present study, we also found that the concentrations of serum IgG4 and IgE were significantly increased in active IgG4-RD patients. Conversely, serum C4 levels were significantly lower in active IgG4-RD patients than in HC. The reduction in serum C4 levels in these active patients may indicate that specific antibodies bind to the target antigen and the activated complement system is common in the disease.

Expanded CD4^+^ CTLs have been reported to play an important role in the pathogenesis of IgG4-RD [4, 20–23]. CD4^+^ CTLs differentiate mainly from Th1 cells. Accordingly, we investigated circulating Th subsets in patients with IgG4-RD. The numbers of circulating Th1 cells were significantly increased in active IgG4-RD patients. In line with our results, increased circulating Th1 cells in IgG4-related sialadenitis patients have already been reported by Ohta et al. and Higashioka et al. [24, 25]. Here, we used surface molecules to characterize the Th subgroup for providing potential diagnostic applications in the future. We also found that the numbers of circulating Th1 cells were well correlated with serum IgG4 levels, which suggests that expanded Th1 cells may be involved in the elevation of serum IgG4. Similarly, Maehara et al. reported that the ratio of CD4^+^ CTLs in lesion tissues from patients with IgG4-related dacryoadenitis and sialoadenitis was positively correlated with serum IgG4 concentrations [4]. Upregulation of Th1 cells or CD4^+^ CTLs will result in the production of more IFN-*γ*, and excessive IFN-*γ* is thought to lead to the pathogenic accumulation of Tfh cells, which in turn leads to the formation of abnormal germinal centers and the production of autoantibodies [26]. This indirect effect may be the explanation for the positive correlation between Th1 cells and IgG4. Besides, we found that the numbers of circulating Th1 cells were positively correlated with the IgG4-RD RI scores. This means that circulating Th1 cells reflect the disease activity of IgG4-RD. The reason may be because the Th1 cells can produce inflammatory cytokines (IFN-*γ*, etc.) and cytolytic molecules (perforin and granzyme), which were associated with the disease activity.

Previous studies investigated the role of Tfh cell subsets in IgG4-RD. However, the results remain controversial. Akiyama et al. reported that activated circulating Tfh1 and Tfh2 cells were increased in IgG4-RD and the number of Tfh2 cells was associated with serum IgG4 levels [27, 28]. Chen et al. reported that frequencies of circulating Tfh1 and Tfh2 cells were significantly increased in IgG4-RD patients [8]. Grados et al. found that circulating T cells polarized toward Th2/Tfh2 and Th17/Tfh17 in patients with IgG4-RD [29]. A recent study showed that all circulating PD-1^+^ Tfh cell subsets were expanded in IgG4-related sclerosing cholangitis and pancreatitis, but only activated Tfh2 cells were associated with disease activity [30]. IL-4^+^ Tfh cells were reported to be significantly increased in secondary lymphoid organs and lesion tissues in IgG4-RD [7], and these IL-4^+^ Tfh cells express BATF, rather than GATA-3, which was identified as a master transcriptional factor of circulating Tfh2 [10]. CXCR3 and CCR6 were found to be upregulated on CD4^+^ Tfh cells in lesion tissues of IgG4-RD patients [4], suggesting that increased Tfh in IgG4-RD might be CXCR3^+^ Tfh and CCR6^+^ Tfh cells. Here, we found that the number of circulating Tfh1 cells was significantly higher in patients with active IgG4-RD. Moreover, the number and proportion of circulating Tfh17 cells were significantly higher in patients with active IgG4-RD than in HC. In contrast, the proportion of circulating Tfh2 cells was significantly decreased in active IgG4-RD patients. Also, the numbers of circulating Tfh1 cells were positively correlated with serum IgG4 levels and IgG4-RD RI scores, which suggests that increased Tfh1 cells may be associated with the elevated IgG4 and might contribute to IgG4-RD.

One of the reasons for the inconsistent conclusions is the different criteria for the inclusion of patients. We included patients in this study who were undergoing glucocorticoid therapy. The changes in Th and Tfh subpopulations in these patients during the treatment process were very different from those of new onset or after treatment. Since most IgG4-RD patients are often in the state of starting treatment, it is difficult to collect new-onset patients, but our results are more of reference significance because it reflects the situation of most patients who come to the hospital. This is also the first time to systematically study the changes of Th and Tfh subsets in patients treated with glucocorticoid. We suspect that glucocorticoid can have an effect on circulating T cell subsets, and changes in T cell subsets can also reflect the effects of treatment.

Based on this, we also conducted a preliminary exploration of the diagnostic value of the Th and Tfh subsets and used ROC curves to evaluate the effectiveness of these subsets for distinguishing the active from remission status of IgG4-RD patients during glucocorticoid treatment. According to our results, the areas under the curve (AUC) were 0.8276 for Th1 and 0.7310 for Tfh1, suggesting that Th1 and Tfh1 numbers could be potential diagnosis makers in monitoring IgG4-RD disease activity. Our research uses flow cytometry to detect molecules on the surface of peripheral blood cells. If it can achieve the purpose of evaluating the treatment of patients, it will have a good application prospect. However, because the sample size is not large enough, it is necessary to expand the sample size in the future to clarify the diagnostic value of Th1 and Tfh1.

In summary, our study demonstrates that active IgG4-RD is characterized by circulating T cell polarization toward Th1/Tfh1 and Th17/Tfh17 during glucocorticoid treatment. Circulating Th1 and Tfh1 levels positively correlate with serum IgG4 levels and disease activity in patients with IgG4-RD, which might play an important role in the course of IgG4-RD, and could be the potential biomarkers for disease activity monitoring in IgG4-RD.

## 4. Materials and Methods

### 4.1. Subjects

A total of 39 patients with IgG4-RD (24 males, 15 females; median age: 62 years) and 22 healthy controls (HC) (13 males, 9 females; median age: 66 years) were recruited from outpatient and inpatient sections of Peking University People's Hospital between December 2018 and May 2019. The diagnosis of IgG4-RD was performed according to the 2011 comprehensive diagnostic criteria [31]. All IgG4-RD patients were receiving glucocorticoid therapy. Most patients have been standardized to dosing: prednisolone 0.6 mg/kg/d for 2–4 weeks and then dose reduction to 5 mg/d after 3-6 months, with the expected cessation of treatment by 2–3 years. The IgG4-RD responder index (RI) score was used for the assessment of disease activity [32]. Each affected organ was scored separately, and all individual organ scores were summed to calculate the overall RI. All RI scores in this present study were calculated with the inclusion of serum IgG4 levels. IgG4-RD RI score ≥ 3 was considered an active disease, and <3 was classified as remission [21]. This research was approved by the Ethics Committee of Peking University People's Hospital and was performed following the ethical standards of the Declaration of Helsinki.

### 4.2. Clinical Indicator Measurement

Serum levels of IgG4 were measured by nephelometry using a Siemens BN II Nephelometer (Siemens Healthcare Diagnostics; Malburg, Germany) and Siemens reagents. Serum concentrations of IgG, C3, and C4 were measured by nephelometry using a Beckman Coulter Immage 800 Nephelometer (Beckman Coulter Ireland Inc.; CA, USA) and Beckman Coulter reagents. CRP in serum was tested by immunoturbidimetry using a Beckman Coulter Chemistry Analyzer AU5800 (Beckman Coulter Ireland Inc.; CA, USA) and Beckman Coulter reagents. Serum IgE levels were tested by a Cobas e601 Electrochemiluminescence Immunoassay Analyzer (Roche; Mannheim, Germany). WBC and lymphocyte counts were determined by Sysmex XE-2100 (TOA Medical Electronics; Kobe, Japan).

### 4.3. Flow Cytometry

Peripheral blood mononuclear cells (PBMCs) were separated by gradient centrifugation with a human lymphocyte separation medium (Dakewei Biotech Co., Ltd.; Shenzhen, China) and then washed twice with PBS. Enriched PBMCs were immediately stained for 30 minutes with the following antibodies: CD3-APC, CD4-PerCP/Cy5.5, CXCR5-APC/Cy7, CXCR3-PE, and CCR6-PE/Cy7. Intracellular staining for Foxp3 was performed using a transcription factor staining buffer kit (Thermo Fisher Scientific-eBioscience; San Diego, CA, USA), according to the manufacturer's instructions. After fixation and permeabilization, cells were incubated with anti-Foxp3 allophycocyanin for 30 minutes. All fluorescent antibodies were purchased from BioLegend (San Diego, CA, USA). Samples were analyzed on FACSCanto using Diva software (BD Biosciences; San Jose, CA, USA). Based on the number of lymphocytes in the complete blood count and the proportion of each subgroup of lymphocytes determined by flow cytometry, the absolute count (per *μ*L) of each subgroup was calculated.

### 4.4. Statistics

Continuous variables are shown as median with 25^th^-75^th^ percentiles. Multiple group comparisons were analyzed using the Kruskal–Wallis test. The Mann–Whitney *U* test was used for comparison between two groups. Also, receiver operating characteristic (ROC) curve analyses were performed to explore the efficiency of parameters in evaluating IgG4-RD disease activity and the AUC values were determined. Statistical significance was determined using GraphPad Prism software V.7.0 (GraphPad Software; San Diego, CA, USA). Statistics with *P* values less than 0.05 were considered to be significant.

## Data Availability

The data used to support the findings of this study may be released upon application to Dr. Changsheng Xia, who can be contacted at xiachangsheng@bjmu.edu.cn.

## Abbreviations

AUC: Area under the curve
FCM: Flow cytometry
IgG4-RD: IgG4-related disease
RI: Responder index
HC: Healthy controls
CTLs: Cytotoxic T lymphocytes
ROC: Receiver operating characteristic
Th: T helper cells
Tfh: Follicular helper T cells
PBMCs: Peripheral blood mononuclear cells
WBC: White blood cell.

## Supplementary Materials

Supplementary Materials Supplementary Figure 1: the levels of serological markers in active IgG4-RD patients, remission IgG4-RD patients, and healthy controls. Supplementary Table 1: clinical characteristics of the patients with IgG4-RD.
