# Supplementary material for: Increased Circulating Th1 and Tfh1 Cell Numbers Are Associated with Disease Activity in Glucocorticoid-Treated Patients with IgG4-Related Disease
Source: J Immunol Res. 2020 Nov 27;2020:3757015. doi: 10.1155/2020/3757015 (PMC7721509; doi:10.1155/2020/3757015)
Supplement: Supplementary Materials — Supplementary Figure 1: the levels of serological markers in active IgG4-RD patients, remission IgG4-RD patients, and healthy controls. Supplementary Table 1: clinical characteristics of the patients with IgG4-RD. [file 3757015.f1.docx]

**Supplementary Files**


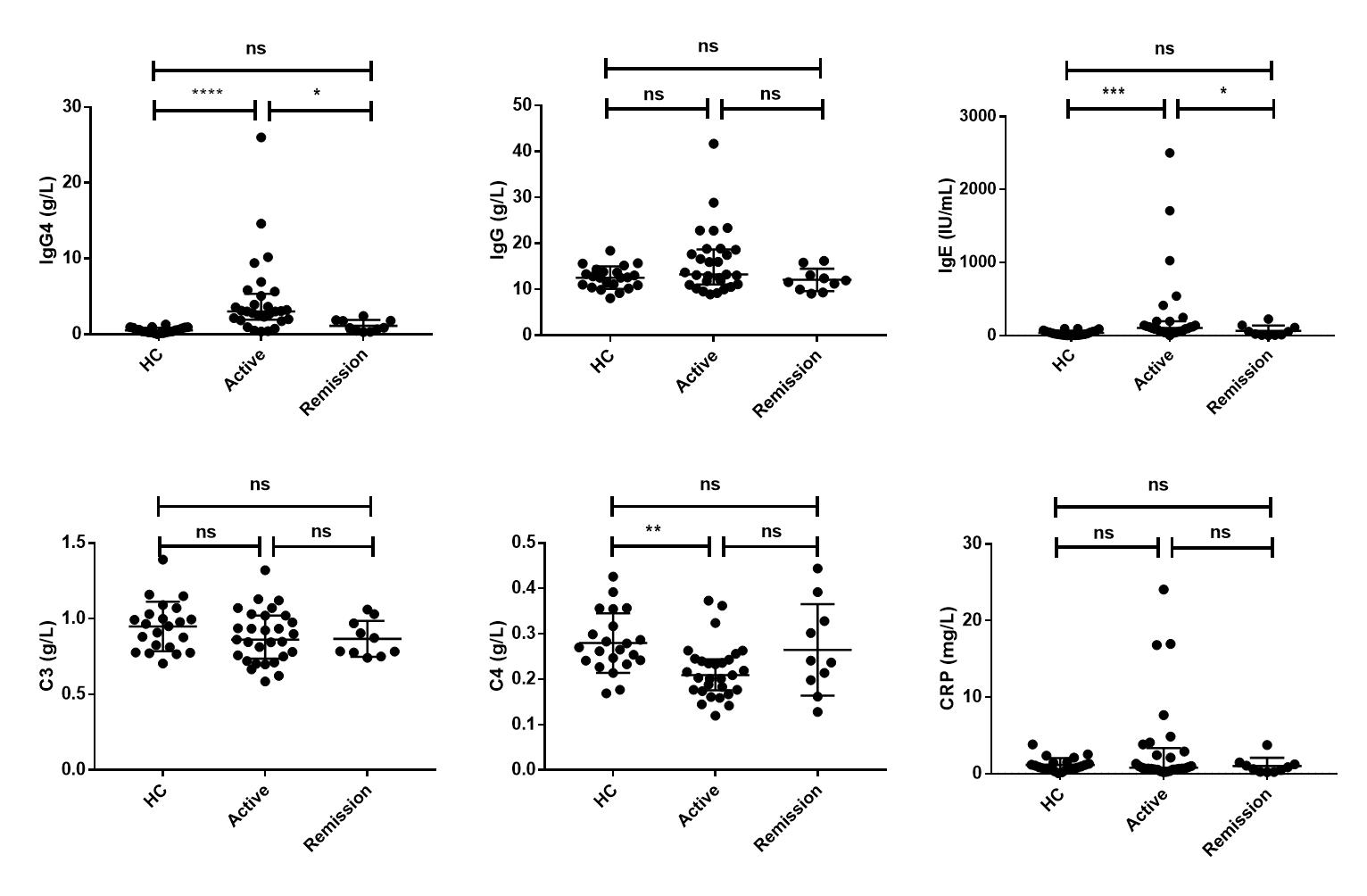


**Supplementary Figure 1. The levels of serological markers in active IgG4-RD patients, remission IgG4-RD patients, and healthy controls.**

The error bars represented the median and inter-quartile range. Data were measured from active IgG4-RD patients (n=29), remission IgG4-RD patients (n=10), and HC(n=22). Levels were compared by Mann–Whitney test; ns, *P* ≥0.05 (not significant); *, *P* <0.05; **, *P* <0.01; ***, *P* <0.001; ****, *P* <0.0001.

**Supplementary Table 1. Clinical characteristics of the patients with IgG4-RD**

| Number | Gender | Age at time of drawing blood, year | Age at diagnosis, year | Age at onset, year | Initial involvement organ | Other involvement organs | Diagnosis | IgG4-RD RI | Glucocorticoid therapy at the time of drawing blood |
| --- | --- | --- | --- | --- | --- | --- | --- | --- | --- |
| 1 | Female | 66 | 60 | 54 | Submandibular gland | Lacrimal gland, parotid gland, sinus | Possible | 10 | Prednisone, 20mg, qd |
| 2 | Female | 65 | 61 | 61 | Submandibular gland | Thyroid, lymph nodes, pancreas | Definite | 6 | Prednisone, 20mg, qd |
| 3 | Male | 76 | 75 | 74 | Lacrimal gland | Submandibular gland, Lung, gallbladder | Definite | 5 | Prednisone, 40mg, qd |
| 4 | Female | 22 | 21 | 20 | Submandibular gland | Lacrimal gland, parotid gland, thyroid, lymph nodes | Definite | 4 | Prednisone, 5mg, qd |
| 5 | Male | 59 | 58 | 56 | Lacrimal gland | Submandibular gland, Lung, pancreas, prostate | Possible | 7 | Prednisone, 40mg, qd |
| 6 | Male | 61 | 59 | 59 | Pancreas | Lacrimal gland, submandibular gland, Lung | Definite | 11 | Prednisone, 15mg, 12.5mg, qod |
| 7 | Male | 49 | 49 | 48 | Pancreas | Lymph nodes, prostate, gallbladder | Possible | 5 | Prednisone, 45mg, qd |
| 8 | Female | 47 | 41 | 38 | Lacrimal gland | Submandibular gland, parotid gland, lymph nodes, liver | Definite | 12 | Prednisone, 2.5mg, qd |
| 9 | Male | 45 | 45 | 41 | Pancreas | Lacrimal gland, submandibular gland, parotid gland | Possible | 6 | Prednisone, 30mg, qd |
| 10 | Male | 64 | 64 | 64 | Pancreas | Lymph nodes | Possible | 7 | Prednisone, 60mg, bid |
| 11 | Female | 40 | 38 | 37 | Submandibular gland | Lacrimal gland, parotid gland | Definite | 8 | Prednisone, 7.5mg, qd |
| 12 | Male | 64 | 61 | 59 | Lacrimal gland | Submandibular gland, parotid gland | Definite | 10 | Prednisone, 15mg, qd |
| 13 | Male | 61 | 54 | 54 | Pancreas | thyroid, Lung, lymph nodes, bile duct, kidney | Possible | 10 | Prednisone, 5mg, 10mg, qod |
| 14 | Male | 54 | 53 | 53 | Lymph nodes | Lacrimal gland, submandibular gland, lung | Definite | 4 | Prednisone, 5mg, qd |
| 15 | Male | 61 | 58 | 45 | Lacrimal gland | Orbital region, submandibular gland, parotid gland, sinus, lymph nodes, prostate, gallbladder, kidney | Definite | 9 | Prednisone, 40mg, qd |
| 16 | Female | 62 | 55 | 49 | Submandibular gland | Lacrimal gland, parotid gland, sinus, thyroid, lung, lymph nodes, heart/ pericardium, pancreas, gallbladder, | Definite | 7 | Prednisone, 20mg, qd |
| 17 | Male | 62 | 55 | 55 | Pancreas | Bile duct, prostate, gallbladder | Possible | 12 | Prednisone, 10mg, qd |
| 18 | Male | 71 | 71 | 69 | Lymph nodes | Lacrimal gland, submandibular gland, sinus, lung, pancreas, kidney | Definite | 11 | Prednisone, 12.5mg, qd |
| 19 | Female | 70 | 68 | 68 | Submandibular gland | Lacrimal gland, parotid gland, pancreas, kidney | Definite | 4 | Prednisone, 5mg, qd |
| 20 | Male | 80 | 79 | 79 | Pancreas | Lacrimal gland, submandibular gland, lung, lymph nodes, bile duct, gallbladder | Possible | 3 | Prednisone, 5mg, qd |
| 21 | Female | 67 | 63 | 63 | Submandibular gland | Sublingual gland, lung, lymph nodes, pancreas | Definite | 16 | Prednisone, 20mg, qd |
| 22 | Male | 58 | 57 | 57 | Submandibular gland | Lacrimal gland, lung, lymph nodes, prostate, gallbladder, kidney | Definite | 6 | Prednisone, 22.5mg, qd |
| 23 | Male | 62 | 62 | 61 | Retroperitoneum | Orbital region, sinus, lung, lymph nodes | Possible | 3 | Prednisone, 12.5mg, qd |
| 24 | Female | 63 | 59 | 49 | Sinus | Lacrimal gland, submandibular gland, lung, gallbladder, kidney | Definite | 6 | Prednisone, 5mg, qd |
| 25 | Male | 69 | 64 | 58 | Lacrimal gland | submandibular gland, parotid gland, sinus, lung, lymph nodes, gallbladder | Possible | 5 | Prednisone, 5mg, qd |
| 26 | Female | 59 | 51 | 38 | Submandibular gland | Orbital region, lacrimal gland, parotid gland, pancreas | Definite | 5 | Prednisone, 30mg, qd |
| 27 | Male | 69 | 63 | 63 | Pancreas | Lacrimal gland, submandibular gland, parotid gland, lung, aorta/ macrovascular | Definite | 4 | Prednisone, 5mg, qd |
| 28 | Male | 77 | 71 | 63 | Retroperitoneum | Lacrimal gland, submandibular gland, parotid gland, lung, lymph nodes | Definite | 6 | Prednisone, 5mg, qd |
| 29 | Male | 72 | 71 | 66 | Parotid gland | Lacrimal gland, submandibular gland, lung, retroperitoneum | Probable | 3 | Prednisone, 12.5mg, qd |
| 30 | Male | 56 | 54 | 54 | Mesentery | Lung, retroperitoneum | Definite | 2 | Prednisone, 5mg, qd |
| 31 | Female | 55 | 51 | 51 | Pancreas | Submandibular gland, parotid gland, lymph nodes, bile duct, gallbladder, kidney | Definite | 2 | Prednisone, 5mg, qd |
| 32 | Female | 38 | 37 | 37 | Submandibular gland | Lacrimal gland, lung, lymph nodes, pancreas | Definite | 2 | Prednisone, 7.5mg, qd |
| 33 | Male | 73 | 73 | 73 | Bile duct | Gallbladder | Possible | 2 | Methylprednisolone, 60mg, qd |
| 34 | Female | 55 | 54 | 53 | Submandibular gland | Lacrimal gland | Definite | 0 | Prednisone, 15mg, 12.5mg, qod |
| 35 | Female | 58 | 55 | 55 | Submandibular gland | Lacrimal gland, thyroid, Lung, lymph nodes | Definite | 1 | Prednisone, 7.5mg, 5mg, qod |
| 36 | Female | 54 | 52 | 52 | Submandibular gland | Lacrimal gland, lung, lymph nodes | Possible | 1 | Prednisone, 10mg, qd |
| 37 | Male | 61 | 61 | 61 | Retroperitoneum | None | Probable | 2 | Prednisone, 22.5mg, qd |
| 38 | Male | 64 | 60 | 60 | Pancreas | Lymph nodes, bile duct | Definite | 2 | Prednisone, 5mg, qd |
| 39 | Male | 64 | 63 | 60 | Lacrimal gland | Submandibular gland, parotid gland, thyroid, lung, lymph nodes, gallbladder | Definite | 2 | Prednisone, 10mg, qd |

Note: IgG4-RD RI, IgG4-related disease Responder Index, calculated among patients with inclusion of the serum IgG4 concentration.
